# New-Onset Atrial Fibrillation Is a Predictor of Subsequent Hyperthyroidism: A Nationwide Cohort Study

**DOI:** 10.1371/journal.pone.0057893

**Published:** 2013-02-28

**Authors:** Christian Selmer, Morten Lock Hansen, Jonas Bjerring Olesen, Charlotte Mérie, Jesper Lindhardsen, Anne-Marie Schjerning Olsen, Jesper Clausager Madsen, Ulla Schmidt, Jens Faber, Peter Riis Hansen, Ole Dyg Pedersen, Christian Torp-Pedersen, Gunnar Hilmar Gislason

**Affiliations:** 1 Department of Cardiology, Gentofte University Hospital, Hellerup, Denmark; 2 Department of Cardiology, Roskilde University Hospital, Roskilde, Denmark; 3 Department of Endocrinology, Herlev University Hospital, Herlev, Denmark; 4 Copenhagen General Practitioners Laboratory, Copenhagen, Denmark; Medical University Innsbruck, Austria

## Abstract

**Aims:**

To examine the long-term risk of hyperthyroidism in patients admitted to hospital with new-onset AF. Hyperthyroidism is a well-known risk factor for atrial fibrillation (AF), but it is unknown whether new-onset AF predicts later-occurring hyperthyroidism.

**Methods and Results:**

All patients admitted with new-onset AF in Denmark from 1997–2009, and their present and subsequent use of anti-thyroid medication was identified by individual-level linkage of nationwide registries. Patients with previous thyroid diagnosis or thyroid medication use were excluded. Development of hyperthyroidism was assessed as initiation of methimazole or propylthiouracil up to a 13-year period. Risk of hyperthyroidism was analysed by Poisson regression models adjusted for important confounders such as amiodarone treatment. Non-AF individuals from the general population served as reference. A total of 145,623 patients with new-onset AF were included (mean age 66.4 years [SD ±13.2] and 55.3% males) of whom 3% (4,620 events; 62.2% women) developed hyperthyroidism in the post-hospitalization period compared to 1% (48,609 events; 82% women) in the general population (n = 3,866,889). In both women and men we found a significantly increased risk of hyperthyroidism associated with new-onset AF compared to individuals in the general population. The highest risk was found in middle-aged men and was consistently increased throughout the 13-year period of observation. The results were confirmed in a substudy analysis of 527,352 patients who had thyroid screening done.

**Conclusion:**

New-onset AF seems to be a predictor of hyperthyroidism. Increased focus on subsequent risk of hyperthyroidism in patients with new-onset AF is warranted.

## Introduction

Atrial fibrillation (AF) is the most common cardiac arrhythmia and a frequent manifestation of hyperthyroidism with a reported prevalence up to 20%, especially in the elderly.[1] Since symptoms of hyperthyroidism are often non-specific and develop slowly, AF may be the first clinical manifestation of thyroid dysfunction. Hyperthyroidism after new-onset AF may not only cause antiarrhythmic treatment failure but also worsen the cardiovascular prognosis.[2], [3] When new-onset AF leads to hospitalization, routine thyroid function testing with measurement of thyroid-stimulating hormone (TSH) is often performed, and even subclinical hyperthyroidism has been associated with a ∼20% increase in cardiovascular mortality.[4], [5] No studies have explored the potential association between new-onset AF and subsequent overt hyperthyroidism. Such an association would suggest the necessity of follow-up assessment of thyroid status after new-onset AF.

To investigate the potential association between new-onset AF and subsequent hyperthyroidism, we conducted a nationwide study comprising over 4 million individuals using individual-level linkage between Danish administrative registries of hospitalization and drug dispensing from pharmacies in the period of 1997 to 2009. Furthermore, a substudy based on biochemically euthyroid subjects (n = 499,689) living in the capital area of Denmark was performed.

## Materials and Methods

### Study Setting

In Denmark, every resident is provided with a permanent and unique civil registration number enabling individual-level-linkage between nationwide administrative registers holding information on health-care usage.[6] Since 1978 the Danish National Patient Registry has registered all hospital admissions in Denmark.[7] Each admission is registered with one primary and, if appropriate, one or more secondary diagnoses using the World Health Organization International Classification of Diseases (ICD). The Danish Register of Medicinal Product Statistics holds information regarding all claimed prescriptions (according to the international Anatomical Therapeutic Chemical (ATC) classification) in Denmark since 1995. The registry also includes information on the date of dispensation, strength, and quantity. All pharmacies are required by Danish legislation to provide information that ensures complete and accurate registration. This registry has been found to be accurate and has been described in more detail previously.[8] Vital status can be obtained from the Central Population Register, which records all deaths within 14 days.[9] Annual incomes for all Danish citizens are registered in the Integral Database for the Danish Labour Market, and socioeconomic status was defined by the individual average yearly gross income in a 5-year period prior to inclusion in the study.[10].

### Population

The study cohort comprised all Danish citizens aged 18 years or more on 1 January 1997, and the cohort was followed until 31 December 2009 or death. We excluded all subjects with previous AF (ICD8 427.93 and 427.94, ICD10 I48), concomitant thyroid dysfunction (defined as previous prescription of L-thyroxine [ATC H03AA01], use of antithyroid drugs [ATC H03B] or established thyroid disease diagnoses [ICD8 240–246, ICD10 E00-E06]), and/or previous usage of amiodarone therapy [ATC C01BD01] due to possible confounding effects in the analysis.

#### General population

All citizens without new-onset AF were defined as the general population.

#### New-onset AF cohort

From the Danish National Patient Registry all citizens with a first-time hospitalization with AF or atrial flutter (ICD10 I48) as primary or secondary diagnosis were identified. All patients alive at discharge were included.[11].

### Co-morbidity and Concomitant Medical Therapy

From the Danish National Patient Registry the following co-morbidities (ICD-8 and ICD-10 codes) were identified: myocardial infarction (410 and I21–22), congestive heart failure (427.0–427.1 and I50), and ischemic stroke (432–438 and I63–66, I69.3, I69.4 and G65). These diagnoses have been validated in the registry with high sensitivity and positive predictive values.[12], [13] Charlson Co-morbidity Index was calculated on basis of pre-specified diagnoses at 1 January 1997 and up to one year previously.[14], [15] From the Danish Register of Medicinal Product Statistics we identified all claimed prescriptions of amiodarone (ATC C01BD01) and antithyroid medication: methimazole (ATC H03BB02), carbimazole (ATC H03BB01), and propylthiouracil (ATC H03BA02). In addition, all prescriptions of beta-blockers (ATC C07), renin-angiotensin system inhibitors (ATC C09A), Vitamin K antagonists (ATC B01AA), loop diuretics (ATC C03C), statins (ATC C10AA), spironolactone (ATC C03DA01), and glucose-lowering drugs (ATC A10) were identified at time of inclusion in the study.[16].

### Outcome

The outcome of interest was onset of hyperthyroidism, which was defined as a first-time prescription of antithyroid medication including methimazole, carbimazole, or propylthiouracil, i.e. drugs that are used exclusively for hyperthyroidism.

### Subgroup Analysis

A subgroup analysis was performed to see if our results were influenced by incident hyperthyroidism at the time of hospitalization with new-onset AF and to characterize the thyroid status at baseline. The analysis was based on data from the Copenhagen General Practitioners Laboratory and departments of clinical biochemistry in three major hospitals in the Copenhagen area. Patients were included who in the period of 1998–2009 had their thyroid function evaluated in-hospital or by their general practitioner using thyroid-stimulating hormone (TSH) and free thyroxine (FT4) plasma levels. TSH and FT4 were determined in serum by the commercially available ADVIA Centaur TSH kit (Bayer/Siemens, Tarrytown, NY) according to the instructions of the manufacturer. In our thyroid assays the normal range for TSH was 0.2–4.0 mIU/L (euthyroidism) and the normal range for FT4 was 9–22 pmol/L. Subclinical hypothyroidism was defined by elevated TSH with normal FT4, and clinical hypothyroidism by elevated TSH with low FT4 or as highly elevated TSH (>10 mIU/L). Subclinical hyperthyroidism was defined by supressed TSH (<0.2 mIU/L) with normal FT4, and clinical hyperthyroidism by supressed TSH with an elevated FT4 or by fully supressed TSH (<0.02 mIU/L). Cases with new-onset AF and normal TSH (measured ±90 days from AF onset) were included. For controls, euthyroid individuals from the background population were included at the time of their first thyroid assay. Follow-up was started 90 days after new-onset AF or at time of first thyroid hormone measurement for the background population. Also, in the substudy, the combined endpoint of antithyroid medication and any hyperthyroid diagnosis was used.

### Statistical Analysis

Baseline characteristics are presented as numbers with percentages for categorical variables and as mean (± standard deviation [SD]) for continuous variables. Categorical variables were shown as proportions, and the differences were analyzed using χ^2^ tests. P values less than 0.05 were considered statistically significant. Time-dependent Poisson-regression models were constructed to derive incidence rate-ratios (IRR) for claiming first-time prescription of anti-thyroid medication and are presented with 95% confidence intervals (CI). The whole population entered the analysis on 1 January 1997 and patients changed status when new-onset AF occurred or antithyroid treatment was initiated. Patients were censored at the end of the follow-up period (31 December 2009) or when they died without reaching an end-point. A 5% significance level was used in all analysis including when testing for interactions. All analyses were stratified by sex and age groups because of significant interaction between sex and age (p<0.0001) and adjusted for amiodarone therapy (time-dependent), calendar year, Charlson Comorbidity Index and socioeconomic status. Normally distributed variables were shown as mean (SD), and differences between groups were analyzed using unpaired t tests. All statistical analyses were performed with the SAS Statistical Software version 9.2 (SAS Institute Inc., Gary, NC, USA) and Stata Software version 11 (StataCorp, College Station, TX, USA).

### Ethics

This study was approved by The Danish Data Protection Agency (ref. 2007-41-1667). Retrospective register studies do not require ethical approval in Denmark. The authors had full access to the data and take full responsibility for its integrity.

## Results

**Figure 1:**
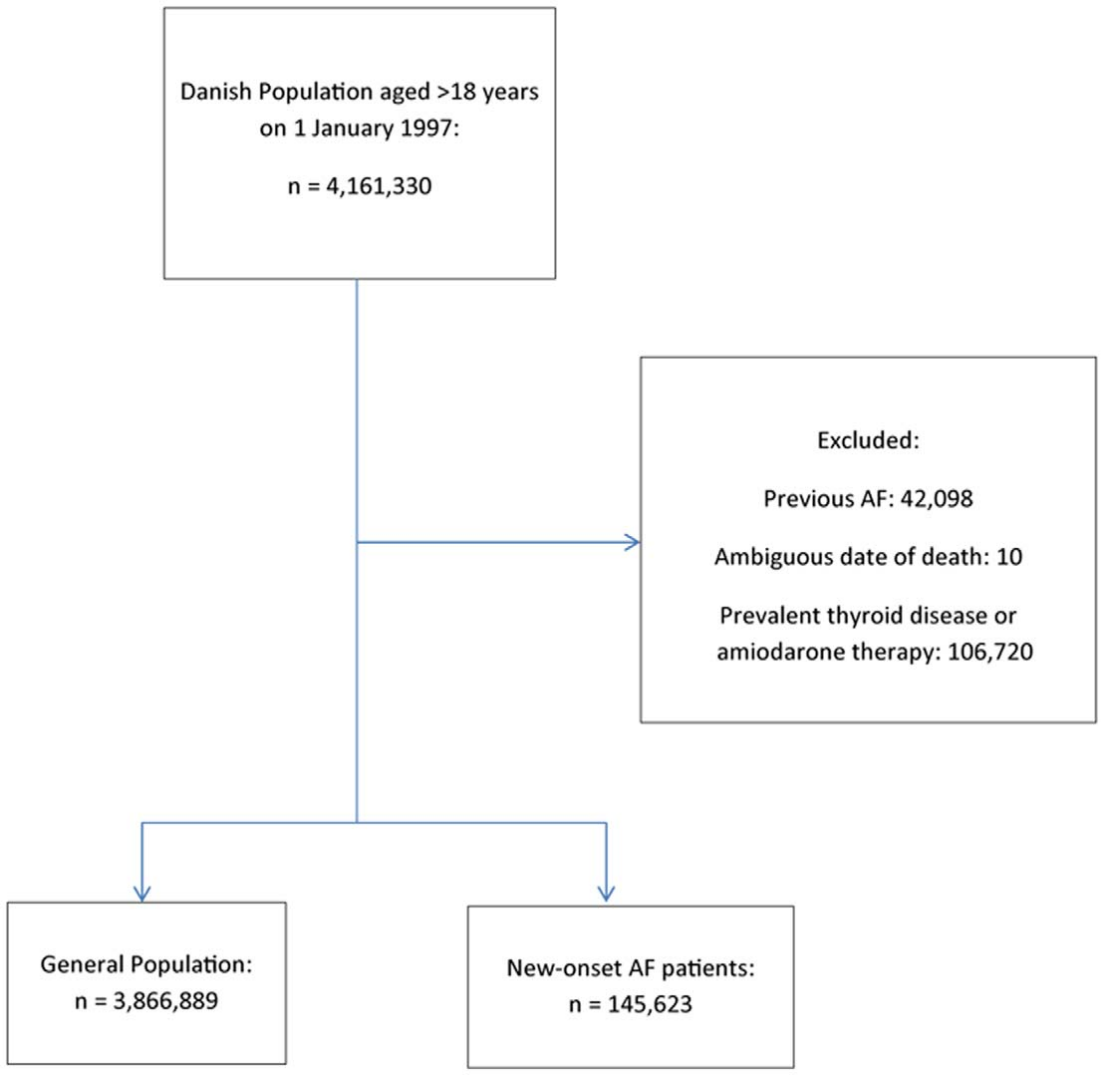
Flowchart of study cohort selection. AF, Atrial Fibrillation.

We identified a total of 145,623 patients from 1997–2009 discharged alive after first-time hospitalization for AF. Selection of the study cohort is depicted in Figure 1. Table 1 shows baseline characteristics for the new-onset AF cohort and the general population (n = 3,866,889). There were slightly more male patients (55.3%) with new-onset AF and they were older and had more comorbidity and concomitant medical treatment compared to the general population.

**Table 1:** Baseline characteristics for the general population and patients with new-onset atrial fibrillation (1 January 1997).

	General Population*(n = 3,866,889)*	New-onset AF cohort*(n = 145,623)*	P value
**Characteristics**			
Women (%)	1,950,730 (50.4)	65,027 (44.6)	<0.0001
Men (%)	1,916,159 (49.6)	80,596 (55.4)	<0.0001
Mean Age, Women (SD)	46.5 (18.5)	71.7 (11.7)	<0.0001
Mean Age, Men (SD)	44.2 (17.0)	64.9 (13.2)	<0.0001
**Comorbidity (%)**			
Peripheral vascular disease	5,118 (0.1)	530 (0.5)	<0.0001
Cerebrovascular disease	11,012 (0.3)	934 (0.8)	<0.0001
Ischemic heart disease	15,515 (0.4)	2,061 (1.8)	<0.0001
Congestive heart failure	6,171 (0.1)	710 (0.6)	<0.0001
Previous myocardial infarction	6,684 (0.2)	1,023 (0.9)	<0.0001
Chronic obstructive pulmonary disease	9,338 (0.2)	962 (0.9)	<0.0001
Cardiac dysrythmia	2,513 (0.1)	605 (0.5)	<0.0001
Renal disease	2,094 (0.0)	192 (0.2)	<0.0001
Cancer	11,775 (0.3)	928 (0.8)	<0.0001
Diabetes mellitus	23,199 (0.5)	1,027 (0.9)	<0.0001
**Treatment (%)**			
Beta-blocker	93,445 (2.1)	9,574 (8.6)	<0.0001
ACE/ARB	90,518 (2.1)	9,091 (8.2)	<0.0001
Vitamin K antagonist	20,511 (0.5)	1,984 (1.8)	<0.0001
Loop diuretic	81,750 (1.9)	9,969 (8.9)	<0.0001
Statin	25,682 (0.6)	1,968 (1.8)	<0.0001
Spironolactone	18,511 (0.4)	1,320 (1.2)	<0.0001
Glucose-lowering drugs	70,665 (1.6)	4,671 (4.2)	<0.0001
**Charlson Comorbidity Index, No. (%)**			
0	3,794,251 (98.1)	139,242 (95.6)	<0.0001
1	39,082 (1.0)	4,155 (2.9)	<0.0001
2	24,982 (0.7)	1,607 (1.1)	<0.0001
3+	8,574 (0.2)	619 (0.4)	<0.0001
**Yearly family income in quintiles, No. (%)**			
0	769,666 (19.9)	32,835 (22.6)	<0.0001
1	759,376 (19.6)	43,128 (29.6)	<0.0001
2	779,877 (20.2)	22,625 (15.5)	<0.0001
3	781,670 (20.3)	20,835 (14.3)	<0.0001
4	776,300 (20.0)	26,200 (18.0)	<0.0001

SD, Standard Deviation; AF, Atrial Fibrillation; ACE, Angiotensin Converting Enzyme Inhibitors; ARB, Angiotensin Receptor Blockers.

### Event Rates for Hyperthyroidism

The total number of patients developing hyperthyroidism as defined by use of antithyroid drugs in the period up to thirteen years (mean follow-up 3.53 years) after new-onset AF was 4,620 (3%) of whom 62.2% were women. In the general population 48,609 (1%) patients developed hyperthyroidism of whom 39,842 (82%) were women.

**Figure 2:**
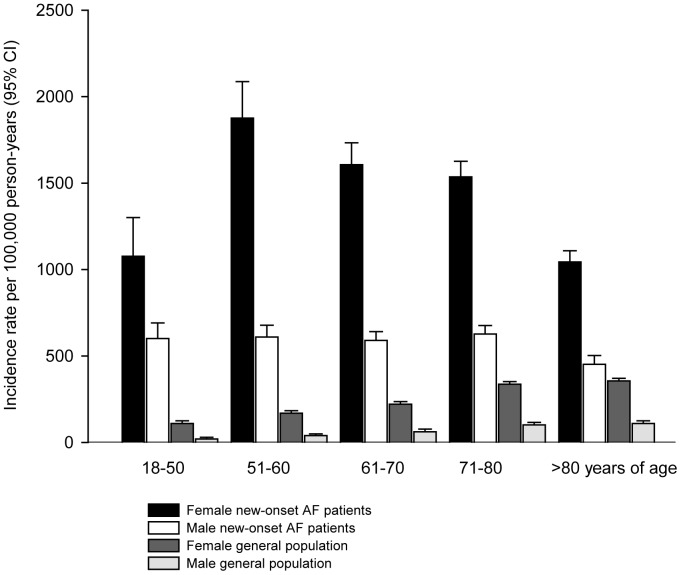
Development of hyperthyroidism in patients with new-onset atrial fibrillation and in the general population stratified by sex and age groups. Incidence rates per 100,000 person-years (95% confidence intervals). AF, Atrial Fibrillation; CI, Confidence Interval.

**Figure 3:**
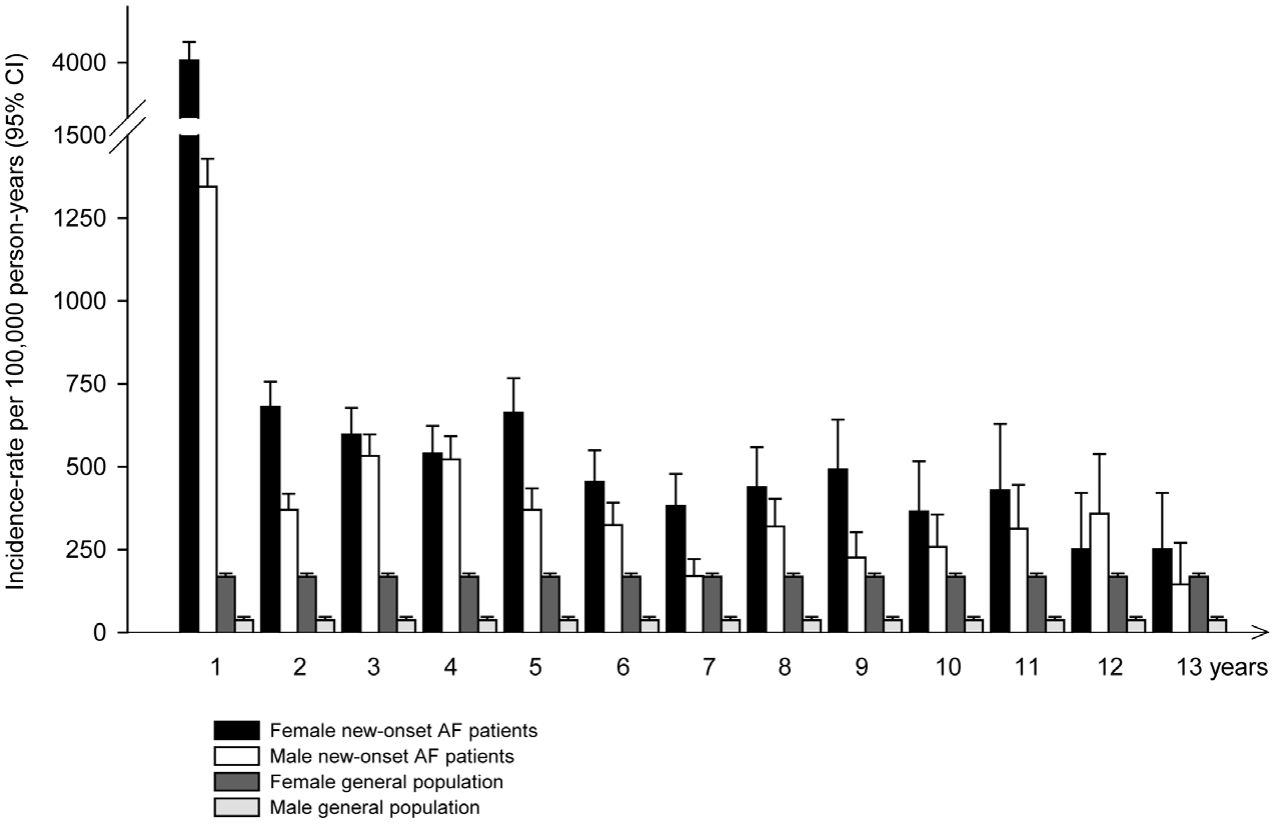
Development of hyperthyroidism in patients with new-onset atrial fibrillation following hospital discharge and in the general population from 1997–2009. Incidence rates per 100,000 person-years (95% confidence intervals). AF, Atrial Fibrillation; CI, Confidence Interval.

Incidence rates for developing hyperthyroidism for the general population and the cohort with new-onset AF are shown in Table 2 and Figure 2. The incidence rates in the general population were on average around 100 per 100,000 person-years as previously described in Danish studies[17], with the highest rates among women and the elderly. The peak rate of incident hyperthyroidism in the new-onset AF cohort was found among women 51–60 years of age, whereas the men had a markedly lower incidence throughout the age groups. The incidence rate of hyperthyroidism was continuously increased following new-onset AF compared to the average rate in the general population (Figure 3). Notably, there was a very high incidence-rate during the initial period after new-onset AF for both women and men.

**Table 2:** Development of hyperthyroidism in patients with new-onset atrial fibrillation and in the general population: observed person-years, numbers of diagnosed patients with hyperthyroidism (events) and incidence-rates (per 100,000 person-years) with 95% confidence intervals (CI) for women and men stratified by age groups.

	*Years of age*	*18–50*	*51–60*	*61–70*	*71–80*	*>80*
**New-onset AF patients**						
*Women*	Incidence rate (95% CI)	1,077 (853–1,358)	1,876 (1,665–2,114)	1,605 (1,478–1,744)	1,536 (1,446–1,631)	1,044 (979–1,115)
	Events	71	270	563	1,054	913
	Person-years	6,596	14,390	35,064	68,640	87,377
*Men*	Incidence rate (95% CI)	601 (511–709)	610 (542–686)	590 (539–645)	627 (578–680)	452 (401–510)
	Events	143	279	476	577	264
	Person-years	23,758	45,731	80,702	92,009	58,367
**General Population**						
*Women*	Incidence rate (95% CI)	110 (109–112)	169 (166–173)	221 (216–226)	337 (329–345)	346 (336–356)
	Events	14,080	7,356	6,826	7,185	4,400
	Person-years	12,751,564	4,342,128	3,089,432	2,132,250	1,272,305
*Men*	Incidence rate (95% CI)	20 (19–20)	40 (39–42)	62 (59–65)	101 (97–106)	110 (102–118)
	Events	2,636	1,841	1,870	1,720	704
	Person-years	13,476,703	4,568,252	3,027,757	1,699,789	641,542

AF, Atrial Fibrillation.

### Risk of Hyperthyroidism

**Figure 4:**
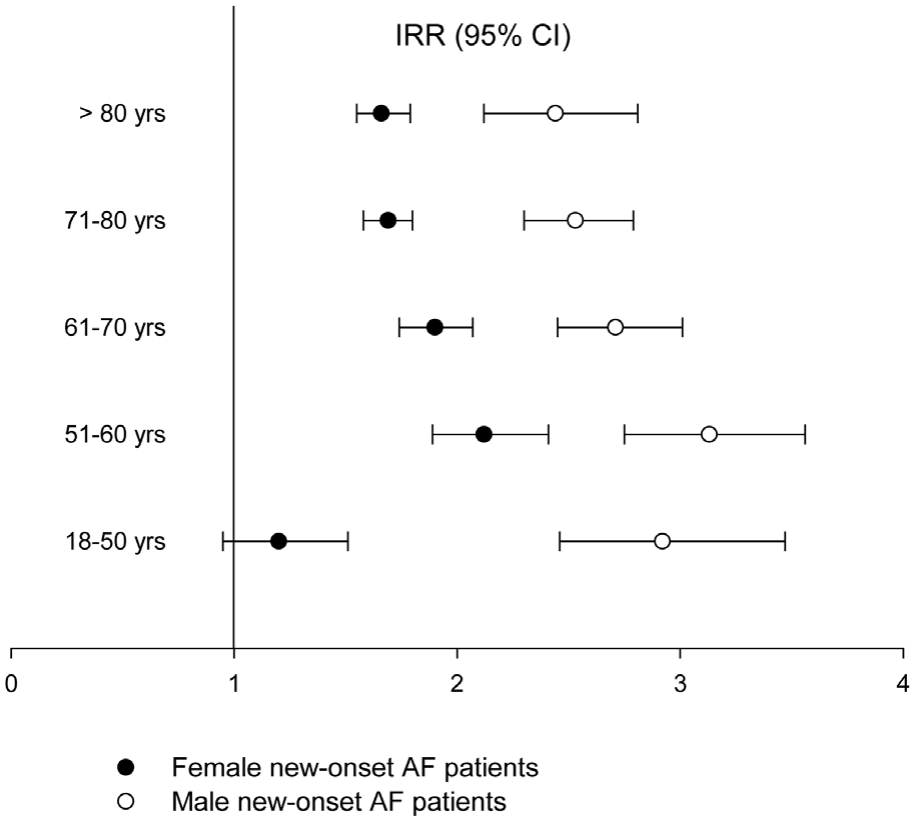
Incidence rate-ratios (IRR) of hyperthyroidism after new-onset atrial fibrillation in women and men stratified by age groups (95% Confidence Intervals, p<0.0001) with the general population used as reference. AF, Atrial Fibrillation; CI, Confidence Interval; IRR, Incidence Rate Ratio; yrs, years.

Age- and gender-stratified multivariate Poisson regression analysis with the general population as reference showed a significantly increased risk of hyperthyroidism in patients with new-onset AF (Figure 4). The highest risk was found in men aged 51–60 years (incidence rate ratio [IRR] 3.31; 95% confidence interval [CI] 2.75–3.56) and the women in the same age-group also had a risk (IRR 2.13 [1.89–2.42]).

### Subgroup Analysis

We identified 527,352 patients (mean age 52.9±17.2 years) with available information on thyroid hormone levels in the period of 1998–2009 (Table 3). In the new-onset AF cohort at baseline, hyperthyroid disease was seen in 318 (2.7%) patients and 701 (5.9%) had hypothyroid disease and where not included in the substudy. A total of 499,689 euthyroid patients were included in the substudy of whom 10,864 patients where euthyroid at the time of new-onset AF (mean age 65.8±13.1 years). During follow-up (mean follow-up 5 years) 1,654 (0.3%) patients were diagnosed with hyperthyroidism, of which 50 (0.5%) were from the new-onset AF cohort and 1,604 (0.3%) from the background population. Overall time at risk was 2,426,322 person-years (32,126 person-years in the new-onset AF cohort). Rates of hyperthyroidism in the euthyroid background population were 93 [88–98] for women and 25 [22]–[28] for men per 100.000 person-years. In the euthyroid new-onset AF cohort the rate was 184 [126–268] for women and 131 [87–198] per 100.000 person-years for men. IRR associated with new-onset AF for developing hyperthyroidism was 1.77 [1.29–2.44] adjusted for sex, age, calendar year, and amiodarone treatment. Using the combined endpoint of thyroid medication and hyperthyroid diagnoses yielded an IRR of 1.78 [1.34–2.36]. Stratifying on sex and adjusting further for Charlson Comorbidity Index and socioeconomic status gave an IRR of 1.33 [0.88–2.01] for women and 3.04 [1.91–4.81] for men.

**Table 3:** Baseline thyroid function from the substudy with individuals who had their thyroid function evaluated (n = 527,352).

	General Population*(n = 515,471)*	New-onset AF cohort*(n = 11,881)*
**Thyroid Function**		
TSH, median [IQR]	1.4 [0.94–2.00]	1.4 [0.88–2.17]
FT4, median [IQR]	14.8 [13.2–16.6]	15.7 [13.6–18.2]
		
Euthyroid	488,831 (94.8)	10,864 (91.4)
Subclinical hypothyroidism	18,954 (3.7)	671 (5.7)
Clinical hypothyroidism	1,039 (0.2)	30 (0.3)
Subclinical hyperthyroidism	5,134 (1.0)	227 (1.9)
Clinical hyperthyroidism	1,513 (0.3)	89 (0.8)

AF, Atrial Fibrillation; IQR, Inter Quartile Range; TSH, Thyroid Stimulating Hormone; FT4, Free thyroxine.

## Discussion

This study demonstrated that one in twenty-five patients admitted with atrial fibrillation (AF) without previous thyroid disease developed hyperthyroidism requiring medical therapy over a period of thirteen years. To our knowledge the present study is the first to demonstrate evidence for such risk. Most importantly, new-onset AF doubled the risk of later occurrence of hyperthyroidism and in particular increased the risk by up to 3-fold in middle-aged men, in comparison with the general population. The importance of new-onset AF as a risk factor for later occurrence of hyperthyroidism was highlighted by a continuously elevated risk throughout the study period.

New-onset AF is a common cause of hospital admission [18] and previous studies have suggested a limited value of in-hospital routine thyroid screening for incident hyperthyroidism in these patients. [19], [20] Results from our subgroup study confirmed that only 0.8% of patients were diagnosed with clinical hyperthyroidism during admission for new-onset AF, whereas a significant number of patients were diagnosed during follow-up. Thyroid screening of new-onset AF patients may well be repeated later on, e.g., at follow-up in the outpatient department or by the general practitioner. This could be of importance as one of the major challenges in AF therapy is the high recurrence rate despite antiarrhythmic therapy both after cardioversion, and among subjects with spontaneous conversion to sinus rhythm. Although several new antiarrhythmic drugs have been developed, the AF recurrence rate remains up to 60% after one year of treatment.[21] This could be because the current treatment of AF is ineffective or because yet unidentified factors promote AF recurrence and latent hyperthyroidism could indeed be such a factor.[22] Also, a recent meta-analysis demonstrates a ∼20% increase in all-cause mortality associated with hyperthyroidism, and although the authors stress that the findings could be caused by e.g. genetic confounding, it does emphasise the possible importance of early diagnosis and timely treatment.[23].

The Canadian Registry of Atrial Fibrillation did not show an increased risk of hyperthyroidism following recent onset AF (<3 months) in a cohort of 707 patient with a follow-up time of 1.7 years.[19] However, this cross-sectional study was designed to assess the value of baseline thyroid function screening in patients with recent onset AF to identify hyperthyroidism, and not the risk of developing hyperthyroidism later on. Not only did we have access to a much larger cohort with a longer follow-up time, but we also did a subgroup analysis using thyroid blood tests (TSH and FT4). Our analysis was also controlled for possible confounders, especially amiodarone therapy [24] which is a known cause of hyperthyroidism due to the high content of iodine. [25].

The substudy confirmed our primary findings in a cohort of euthyroid patients where follow-up was postponed to 90 days after new-onset AF to isolate later-onset hyperthyroidism. Rates of developing hyperthyroidism were generally lower in the substudy, though still two-fold higher for women and five-fold higher for men in the euthyroid new-onset AF cohort. This lower rate is partly due to exclusion of prevalent hyperthyroid cases and the smaller sample size, but might also be because of regional differences in iodine intake in Denmark, with a higher intake in the Copenhagen area from where our substudy cohort was taken.[26] The overall risk was ∼80% higher for developing hyperthyroidism in the euthyroid new-onset AF cohort and was significantly three-fold higher for the men, but, interestingly, the risk was only ∼30% higher for women in the substudy (not significant). Analyses using a combined endpoint of antithyroid medication and hyperthyroid diagnosis did not considerably alter these risk-estimates.

Although we did not have access to data on thyroid autoantibodies in the present cohort of patients, recent studies have demonstrated that circulating activating autoantibodies often found in patients with Graves’ hyperthyroidism can facilitate development of AF. This could contribute to our results as β1-adrenergic and M2-muscarinic receptor autoantibodies may trigger development of AF before thyroid dysfunction with abnormal thyroid hormone levels becomes evident.[27] An intriguing possibility is that genetic susceptibility to AF could in itself be linked to hyperthyroidism.[28] However, it is most likely that our findings are explained, in part, by high-normal thyroid function that is known to alter central hemodynamics[29] and to be arrhythmogenic.[30] Furthermore, the normal reference interval for thyroid function for an individual is known to be more narrow than the general laboratory reference intervals.[31] Therefore, our findings could also be explained by variations in thyroid function within the individual patient, that are not detected in view of the wider reference range for the general population. Thus, we speculate that discrete changes in thyroid function in an individual reflected by a relative fall in TSH or an increase in FT4 within the normal laboratory reference intervals, but below the normal range for this particular subject, could trigger onset of AF.[32], [33] In such cases, a diagnosis of hyperthyroidism would not be made at the time of hospitalization with AF, but instead at a later time, when hyperthyroid disease becomes clinically and/or biochemically evident.

### Strengths and Limitations of the Study

The main strength of the study was the nationwide cohort of unselected patients with AF. The size of the population solidified the results and absence of selection bias enabled us to extrapolate the results to a wider population of patients. Another important fact is that antithyroid treatment is specific for hyperthyroidism, leaving little doubt about the robustness of our primary outcome. Furthermore, none of the drugs studied are available over the counter in Denmark.

There are several limitations to the study that need to be acknowledged. Being an observational study it is not possible to draw direct conclusions on potential causal relationships of the findings and the results can be a result of bias and confounding. Specifically, it was impossible to explore the reasons for the individual patients’ start of antithyroid therapy. One can speculate that patients previously admitted with new-onset AF are more prone to subsequently consult their general practitioner and have their thyroid function evaluated over time (i.e., implicating surveillance bias of our results). Furthermore, the study was based on administrative registers that do not include clinical parameters correlated to outcome such as body mass index, smoking status, lipid levels, thyroid autoantibodies, or electrocardiogram findings. Neither did we have data on the types of hyperthyroidism, e.g., Graves’ disease or nodular disease.[34] Hyperthyroidism was defined as use of antithyroid drugs and this might underestimate the true incidence of hyperthyroidism since patients with subclinical hyperthyroidism usually have a multinodular goitre and are typically treated with radioiodine in Denmark without pre-treatment with antithyroid drugs. These patients were not registered in our study, but we have no reason to believe that this possible underestimation of the hyperthyroidism incidence was different in the new-onset AF cohort and general population.

We were unable to detect patients with first-time AF who were not hospitalized, but treated by their general practitioner, which could lead to information bias. However, most patients diagnosed with AF in Denmark will have a hospital admission or contact with an outpatient clinic and the AF diagnosis has both a high sensitivity and specificity in the Danish registers.[35], [36] There will of cause be some patients in the general population group with undiagnosed AF, and this might cause a slight underestimation of the hyperthyroidism incidence.

Although we have controlled for amiodarone treatment we cannot rule out that other factors related to treatment of AF could be the cause of later occurring hyperthyroidism (i.e., protopathic bias). The Danish population comprises mainly Caucasians and therefore application of these results to other ethnic groups should only be done with care. Finally we were unable to obtain a complete set of laboratory data on thyroid hormone function, apart from the population included in the subgroup analysis.

### Clinical Implications

Our findings are important because they demonstrate that patients with new onset AF are more prone to develop hyperthyroidism during long-term follow-up. The mechanism behind this observation remains unknown. It is well known that patients with hyperthyroidism should be screened for AF, but our study suggests that patients with new-onset AF, without previous thyroid disease, should have routine follow-up examination of their thyroid function. However, this study does not allow us to recommend a specific interval for thyroid screening. Several recent register studies have shown that approximately 10% of AF patients have thyroid dysfunction.[37]–[39] Our results indicate that additional 3% will develop overt hyperthyroidism after new-onset AF. Thus, a large number of AF patients may subsequently suffer from hyperthyroidism that not only may complicate the management of AF but also worsen the overall prognosis for the patient.

### Conclusion

In this nationwide study, patients with new-onset AF had a twofold-increased risk of hyperthyroidism over a 13-year period and the risk was substantially increased especially among young males. Increased focus on development of hyperthyroidism during long-term follow-up in patients with new-onset AF and investigations of the potential value of follow-up assessment of thyroid status in these patients is warranted.
